# Patterns of infection and impact on outcome in haematology patients admitted to intensive care

**DOI:** 10.1186/cc9916

**Published:** 2011-03-11

**Authors:** R José, I McDonald, P Pfeffer, S Shaw, C Kibbler, B Agarwal

**Affiliations:** 1 Royal Free Hospital, London, UK

## Introduction

Infections with opportunistic pathogens in stable chronic haematological patients are well known. Recent reports suggest that these patients admitted to intensive care (ICU) tend to do as well as or better than those without infection [1]. We sought to study the pattern of all infections diagnosed in haematology patients in our ICU.

## Methods

Data on infections were retrospectively collected for haematology patients consecutively admitted to our unit (tertiary haematology referral centre) for the period of January 2005 to December 2008. Readmissions (9/106) were excluded. Bacteria, mycobacteria and fungi were identified by culture and viruses detected by DNA PCR. Coagulase-negative staphylococcus was excluded from the analysis, as they most probably represented contaminants. Data were analysed with SPSS software.

## Results

Ninety-seven patients were admitted during the study period, 71% with known or clinically suspected infection. The most commonly identified bacteria were *Pseudomonas aeruginosa *(15.4%) and *Enterococcus faecalis *(11.3%); viruses were cytomegalovirus (CMV) (17.5%) and respiratory syncytial virus (RSV) (17.5%); and fungi were Candida species (6.2%). Known or clinically suspected infection at admission, identifying an organism, presence of infection with multiple organisms, and infection type were not associated with increased ICU or hospital mortality (*P *> 0.05), but resulted in significantly longer ICU and hospital LOS. Increased ICU LOS (days) (mean (SD)) was associated with identifying an organism (7 (8) vs. 16 (6); *P *< 0.001), number of organisms per patient (0, 1, 2, 3) (7 (7), 13 (13), 16 (8), 41 (29); *P *= 0.006), infection type (not identified, bacterial, viral, mixed, fungal) (7 (8), 15 (19), 16 (12), 17 (9), 26 (24); *P *< 0.001)), viral infection (11 (15), 16 (11); *P *= 0.005), CMV viraemia (11 (14), 18 (12); *P *= 0.002), while increased hospital LOS (days) (mean (SD)) was associated with identifying an organism (37 (34) vs. 61 (60); *P *= 0.004) and infection type (not identified, viral, fungal, bacterial, mixed) (37 (34), 47 (34), 52 (41), 67 (77), 69 (36); *P *= 0.025).

## Conclusions

Most patients with haematological diagnoses admitted to our ICU had a clinically suspected or documented infectious cause. Although infection characteristics are not associated with overall mortality, they are associated with prolonged ICU and hospital LOS.
